# Implementation contexts and strategies for alternative peripherally inserted central catheter material and design selection: A qualitative exploration using CFIR/ERIC approach

**DOI:** 10.1111/jan.16342

**Published:** 2024-07-24

**Authors:** Deanne August, Rachel M. Walker, Victoria Gibson, Nicole Marsh, Tricia M. Kleidon, Alana Delaforce, Claire Mihalopoulous, Amanda Ullman, Samantha Keogh

**Affiliations:** ^1^ School of Nursing, Midwifery and Social Work University of Queensland St Lucia Queensland Australia; ^2^ Nursing and Midwifery Research Centre Royal Brisbane and Women's Hospital Herston Queensland Australia; ^3^ School of Nursing and Midwifery Griffith University Meadowbrook Queensland Australia; ^4^ Division of Surgery Princess Alexandra Hospital Woolloongabba Queensland Australia; ^5^ Children's Health Queensland Hospital and Health Service South Brisbane Queensland Australia; ^6^ School of Nursing Queensland University of Technology Kelvin Grove Queensland Australia; ^7^ Australian e‐Health Research Centre Commonwealth Scientific and Industrial Research Organisation (CSIRO) Herston Queensland Australia

**Keywords:** Consolidated Framework for Implementation Research, contexts and strategies, design key opinion leaders, Expert Recommendations for Implementing Change, implementation, infection, insertion, material, peripherally inserted central catheter, selection, thrombosis

## Abstract

**Aim:**

To explore the implementation contexts and strategies that influence the uptake and selection of alternative peripherally inserted central catheter (PICC) materials and design.

**Design:**

Qualitative evaluation of end user perspectives within a randomized control trial of different PICC materials and design.

**Methods:**

Semi‐structured interviews with key stakeholders were undertaken via an adapted, rapid‐analytic approach using the Consolidated Framework for Implementation Research. Outcomes were mapped against the Expert Recommendations for Implementing Change (ERIC) tool for strategies to guide innovation in PICC practice.

**Results:**

Participants (*n* = 23) represented a combination of users and inserters/purchasers, from adult and paediatric settings. Dominant themes included intervention characteristics (intervention source), inner setting (structural characteristics) and individuals involved (self‐efficacy). Strategies emerging to support a change from ERIC mapping (*n* = 16) included promotion of intervention adaptability, inclusion of staff and consumer perspectives and sufficient funding. Implementation contexts such as inner setting and individuals involved equally impacted PICC success and implementation effectiveness and enabled a greater understanding of barriers and facilitators to intervention implementation in this trial.

**Conclusion:**

Trial evidence is important, but healthcare decision‐making requires consideration of local contexts especially resourcing. Implementation contexts for Australian healthcare settings include a practical, strategic toolkit for the implementation of alternative PICC materials and designs.

**Reporting Method:**

This study adhered to COREQ guidelines.

**Patient or Public Contribution:**

No patient or public contribution.

**Impact**

**What problem did the study address?**

The barriers and enablers to innovation in peripherally inserted central catheter (PICC) selection and practice.

**What were the main findings?**

Key barriers included: focus on service demands, PICC design/agility, alternative vascular access device (tunnelled central venous catheter), followed by PICC material (antimicrobial/hydrophobic) and product familiarity. Strategies to overcome these would require working with industry to consider adaptable options to meet clinician/consumer needs and appropriate resources to initiate and sustain change.

**Implications for the profession and/or patient care**

PICC selection is tied to product familiarity, influenced by historical preferences and individual beliefs. Future studies should focus on patient perspectives.

## 1 INTRODUCTION

Peripherally inserted central catheters (PICCs) are one of the most frequently used vascular access devices selected to deliver medical therapies, yet between 22% and 39% develop complications such as occlusion, deep vein thrombosis or infection (Chopra et al., 2022; Kleidon et al., 2021). Performance of these devices can alter the delivery of essential, moderate or long‐term treatments such as antibiotics and chemotherapy. This poor performance and sequelae have provoked several generations of device innovation since 1970 (Ullman et al., 2019). While vascular access device utilization and selection decisions can be evidence‐based and embedded into clinical practices (Chopra et al., 2015; Ullman et al., 2020); PICC material choice is less understood. Over the past 20 years, there has been an increase in the frequency of PICC use and more recently the variety of choice (material and design) (Chopra et al., 2015; Gorski et al., 2021). This popularity is related to PICC benefits including peripheral insertion (compared to central insertion), emergence of vascular access teams or embedded PICC insertion services (Carr et al., 2018; Schults et al., 2019) and ability to place PICCs at the patient's bedside rather than in procedural settings (Ullman et al., 2019). Interdisciplinary teams often manage and use PICCs, yet nurses are the largest workforce that insert, manage and/or conduct surveillance of PICC function or failure (Chopra et al., 2017). This places nurses in a unique position to advocate for and select ideal PICC material for individual and specific patient populations. However, there is a paucity of evidence to understand what influences decision‐making for PICC choices (material and design) and hospital policy. Drivers of decision‐making can be better understood through the use of implementation science theory such as the Consolidated Framework for Implementation Research (CFIR) (Damschroder et al., 2009). Yet, recent studies have demonstrated infrequent utilization of implementation theories within vascular access research (Comber et al., 2025; Xu et al., 2023). This study rigorously applies implementation science frameworks to identify barriers to best practice (CFIR; Damschroder et al., 2009) and strategies to not only address them but also enhance what is currently working in practice as evidenced or suggested by the Expert Recommendations for Implementing Change (ERIC) (Powell et al., 2015).

## 2 BACKGROUND

The popularity of PICCs has prompted the integration of innovative modifications to traditional polyurethane PICCs to improve function and minimize the risk of complications and device failure (Chopra et al., 2013; Ullman et al., 2017, 2022). The two current methods of modifying polyurethane PICCs include coating/impregnating with antibiotic or antimicrobial agents (e.g. chlorhexidine) (Ullman et al., 2022) or integrating hydrophilic/hydrophobic materials into the base polyurethane to prevent thrombus adherence/accumulation (Ullman et al., 2019). Despite these differences, PICCs have similar characteristics including injectable capacity, priming volume and length, with only the external surface of the catheter colour distinguishing products for unfamiliar clinicians. However, prior comparative research between PICCs is limited to primarily observational studies (Schults et al., 2019) with little exploration surrounding clinicians' impressions and experiences on how these devices function in real clinical settings.

The clinical and cost‐effectiveness of three different PICC materials were evaluated in a randomized control trial (RCT) comparing (i) hydrophobic, (ii) antimicrobial and (iii) polyurethane PICCs in paediatric and adult patients (Ullman et al., 2021). At the onset of the RCT, little was known about clinicians' beliefs and perceptions about different materials, or considerations regarding the interaction of PICC material and patient or device outcomes. As impressions of PICC success and failure were contingent on a broad range of users; including, but not limited to inserting clinicians and PICC users/purchasers (Kleidon et al., 2018), understanding the fit of an innovation/intervention (e.g. PICC material) into the specific needs of these users was vital (Powell et al., 2015). More specifically, factors influencing the acceptance of alternative PICC materials and design (those that include hydrophobic or antimicrobial properties versus polyurethane without special/additional properties) required exploration via an implementation theory lens, effective for the healthcare setting. The CFIR was chosen based on its systematic approach to predicting or explaining barriers and facilitators (determinants) to implementation effectiveness (Damschroder et al., 2022). In addition, the ERIC tool was developed from expert consensus for implementation strategies likely to influence CFIR constructs and support understanding for maximal change (Powell et al., 2015).

## 3 THE STUDY

### 3.1 Aim

This study aimed to explore the implementation contexts and strategies that influence the uptake and selection of PICC innovation, using deductive analysis informed by the CFIR domains and constructs and mapped against the ERIC tool to identify strategies that may assist in enhancing the uptake of changing or selecting between PICC materials.

### 3.2 Design

A qualitative evaluation was undertaken in parallel to a clinical trial, to formally explore potential and actual influences surrounding the implementation of different PICC materials use and selection across the three metropolitan, quaternary hospitals. The 2009 CFIR (Damschroder et al., 2009), adapted rapid analytic approach (Gale et al., 2019), was utilized to identify barriers and enablers to potential practice change and map these against the ERIC tool (Level 1 strategies). (Powell et al., 2015; Waltz et al., 2019) Barriers and enablers then informed an implementation enhancement plan for implementation of alternative PICC material choice(s). This study adhered to the COnsolidated criteria for REporting Qualitative research (COREQ) (Tong et al., 2007) reporting guidelines. To assist the non‐specialist vascular access nurses or clinicians with the content of this study, a Glossary of Vascular Access Terms is provided in Table S1.

### 3.3 Setting and recruitment

The study included three quaternary hospitals within a metropolitan centre of one Australian State. The health services are all within a 10‐km radius and include the Princess Alexandra Hospital (Metro South Health Service), the Royal Brisbane and Women's Hospital (Metro North Hospital and Health Service) and the Queensland Children's Hospital (Children's Health and Hospital Service). Within these hospitals, eight departments were directly involved in PICC insertion, management and purchasing, with additional auxiliary departments (including inpatient and outpatient) involved in the management of PICCs. All hospitals that used one of the study interventions as standard practice and had exposure to the second PICC material (hydrophobic PICC) in recent pilot studies or product trials (Kleidon et al., 2018).

Within these hospitals, there were three different PICC insertion models established. Two sites had a PICC insertion service that included a combination of (i) advanced practice, nurse‐led at bedside, nurse‐led in the department of imaging and (ii) department of imaging PICC insertion service. The third site had only one PICC insertion service that was situated within a department of imaging. One hospital was a dedicated paediatric service, with the other two dedicated adult services both of which provided PICC insertions to adults with the occasional older paediatric patient (15–17 years). PICC insertions had been occurring within all three hospitals for between 9 and 30 years, and they were the lead sites for PICC insertion services across their respective healthcare districts (encompassing over 12 hospitals). All hospitals provided central line‐associated bloodstream infection (CLABSI) surveillance, with two hospitals incorporating other PICC outcome(s) (thrombosis into performance indicators for the vascular access teams).

### 3.4 Population, participants and recruitment

A purposive sampling approach was used to invite representative individuals (50 across three sites) from three stakeholder groups to ensure representation from key areas of PICC practice (PICC inserters, users and purchasers) and a range of experience levels (frequent and infrequent users or inserters) to participate in individual interviews. Interdisciplinary members (doctors and nurses) were included who were from stakeholder groups and represented nurse practitioners, anaesthetists, unit managers, surveillance teams, department heads and radiologists. Students and clinicians not involved with PICC were not invited to participate. Interviews were conducted until data sufficiency was achieved (LaDonna et al., 2021).

The investigation team distributed a study information summary via hospital email to encourage potential participants to contact the investigators and Research Nurses at each site. Following contact with participants, and prior to the interview, informed consent was achieved. The research team used the 2009 CFIR domains (i.e. intervention/individual characteristics, inner setting, outer setting and implementation process) and practice guidelines (Curtis & Ray‐Barruel, 2021; Gorski et al., 2021) to develop semi‐structured interview questions (see Table 1 Interview Guide). Using this guide, semi‐structured interviews were used to explore the importance of PICC selection and identify and assess potential barriers and facilitators for implementation of different PICC innovations (material and design) and interviews conducted by members of the team outside direct supervision or team structures (D.A., R.M.W., V.G. and C.M.). Timing of the interviews was conducted prior to the completion of RCT recruitment to capture clinician familiarity with the intervention. Within this paper, formatting has been used to support the reader's identification of CFIR items; domains are bolded with constructs are italicized and underlined.

**TABLE 1:** Interview guide with related CFIR construct.

CFIR construct	Interview questions		
	PICC inserter	PICC user	PICC purchaser
General introduction	What are your overall thoughts about PICC insertion and use at this hospital? Have you been involved, or are you aware of the recent trial, PICNIC?	What are your overall thoughts about PICC insertion and use at this hospital? Have you been involved, or are you aware of the recent trial, PICNIC?	What are your overall thoughts about PICC selection and utilization at this hospital? Are you aware of the recent trial, PICNIC?
2 Intervention characteristics Evidence strength and quality Relative advantage	What do you need to make an informed, evidence‐based decision to select the most appropriate PICC to insert for each specific patient/circumstance? What are the considerations informing which PICC to select to use?	What are the factors that should be considered in the selection of type of PICC used? Do you believe that there should be a change in practice in PICC utilization?	What information do you need to make an informed, clinically appropriate decision in relation to the type of PICCs to be purchased for use? Do you believe that there should be a change in practice in PICC procurement?
3 Outer setting Patient needs and resources	What patient‐related factors influence which type of PICC you select to use?	What patient‐related factors should be assessed and considered to inform PICC selection?	What do you feel we should consider in using the most appropriate PICC?
4 Inner setting Tension for change Relative priority Leadership engagement	Are there any issues with continuing with the current practice in PICC selection? Is it important that we change practice in line with recent evidence in relation to PICC selection? How can leadership support a change in practice?	Are there any issues with continuing with the current practice in PICC selection? Is it important that we change practice in line with recent evidence in relation to PICC selection? How can leadership support a change in practice?	Are there any issues with continuing with the current practice in PICC selection? Is it important that we change practice in line with recent evidence in relation to PICC selection? How can leadership support a change in practice?
5 Characteristics of individuals Knowledge and beliefs about intervention	How do you feel about a change in practice in relation to PICC selection?	How do you feel about a change in practice in relation to PICC selection?	What do you believe is the most important factor in PICC selection?
6 Implementation process Planning Opinion leaders	What steps do we need to take to successfully implement a change in practice? Who is most appropriate to lead this change in practice?	What steps do we need to take to successfully implement a change in practice? Who is most appropriate to lead this change in practice?	What steps do we need to take to successfully implement a change in practice? Who is most appropriate to lead this change in practice?

Abbreviations: CFIR, Consolidated Framework for Implementation Research; PICC, peripherally inserted central catheter.

### 3.5 Intervention

In 2019, a comparative effectiveness RCT commenced, comparing three PICC materials targeting the prevention of thrombosis and infection. Despite differences in PICC innovation (material and/or minor design variations such as valve compared to clamp) the three PICCs had similar: power injection capacity (4–5 ml/s), priming volume (<1 mL), open tip, length (50–55 cm), diameter (differences less than 0.2 m) and required external needleless connectors. While not impacting PICC function, each device had minor design variations including unique colouring of external tubing and a different catheter hub (with an imbedded anti‐reflux valve). Two of the three PICCs had identical supplementary insertion kits and industry recommendations for insertion, and all three sites used at least one of the study PICCs as standard practice and/or had recently conducted a local product trial evaluation and/or piloted RCT with the third PICC material (Kleidon et al., 2018).

As the RCT considered comparative effectiveness, in terms of standard care and alternative materials (Ullman et al., 2021), the study PICCS was mentioned as a familiarization step at the commencement of the interview, with broad or no material type levels prompted by interview guide, leading the participants to discuss material based on their impression and experience.

### 3.6 Data collection

Individual interviews were conducted via online meetings in spaces determined by interviewees. One of the investigators (R.M.W., D.A., V.G. and C.M.) moderated the sessions, while a second researcher took field notes against the interview guide. Moderation enabled review of research objectives and confirmation of participant consent for participation and audio/video recording. Participants were given the opportunity to ask questions or clarify their understanding of the processes. An interview was considered complete when key points from the interview guide had been discussed and participants had no further comments (Malterud et al., 2016) (Table S2).

Interviews were spread across a number of weeks to allow for a review of transcripts between sessions. Research team meetings and discussions were held at regular intervals between sessions, to review initial responses and identify interim and final themes in line with Gale and colleagues' rapid analytic approach (2019) (Ullman et al., 2022). Member checking was not conducted as individual participant's identities remained anonymous and confidential. Following interviews, de‐identified recordings were transcribed by an external transcription service. Any identifiable participant characteristics, locations or references were removed from the transcripts. Data collected during this study were treated confidentially; coded transcripts and notes were safely stored with the university files along with consent forms.

### 3.7 Data analysis

The analysis comprised of six steps and employed a modified rapid analytic method derived from Gale et al. (2019). The rapid analytic approach bypassed codebook development and instead focused on using simple tables for researchers to extract data. For example, rather than coding by lines of text and circulating for agreement, researchers considered what aspects of the interview applied to the relevant summary table column, for example, *outer setting*, and then entered the statement(s) from the interview into the relevant space.

**FIGURE 1:**
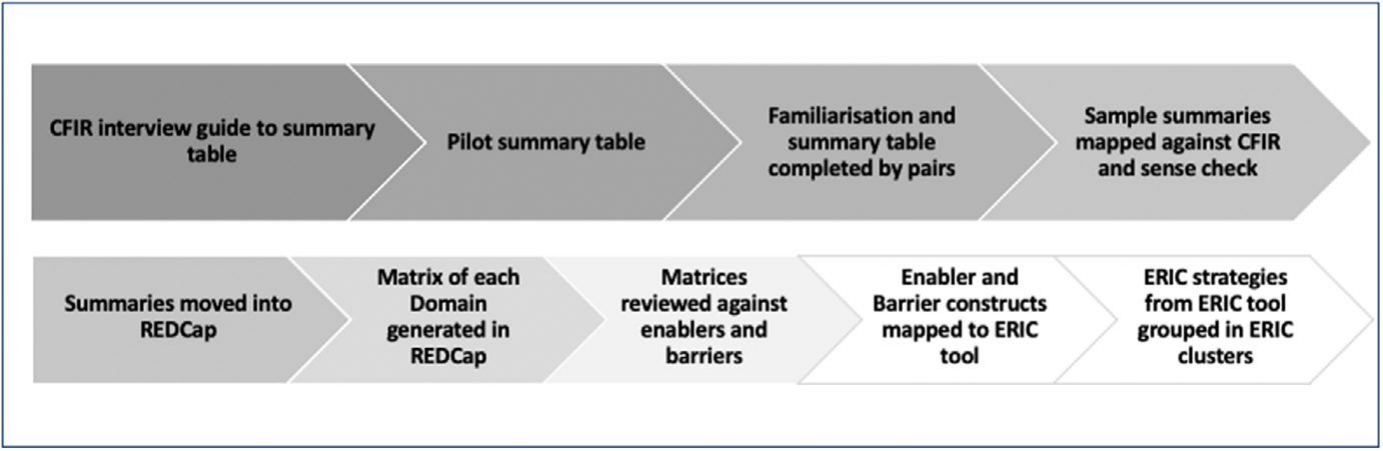
Analysis processes. CFIR, Consolidated Framework for Implementation Research; ERIC, Expert Recommendations for Implementation Change.

Firstly, the CFIR‐based interview guide (Table 1) was used to develop a summary table (Table S2; Figure 1) verbatim transcripts were abridged and then independently piloted by investigation members using a random set of transcripts (D.A., V.G., R.M.W. and S.K.). The first column of the summary table listed the pre‐specified CFIR domains based on the interview guide, with the second column listing descriptive quotes from interviews.

In the second step, the investigation team undertook familiarization with the transcripts in independent pairs (R.M.W., V.G., T.K., S.K., A.U., N.M.) and populated the summary tables. This was followed by an in‐person team meeting where a selection of pilot transcripts were mapped against the domains and constructs, for consensus checking. The independent pairs then reviewed sets of transcripts (seven each), according to CFIR domain and construct, to identify the supporting statement and subconstruct and provide an exemplar quote (Table 2). These summaries were then transferred verbatim into an electronic tool (REDCap) per construct/domain. A matrix for each domain was generated by experts in qualitative evaluation (D.A. and S.K.) who independently coded fragments of text to decide if they represented a barrier or enabler to PICC implementation based on material selection and use (Delaforce et al., 2023). A final meeting of the entire investigation team revised and confirmed enabler and barrier statement(s) until consensus was achieved (Gale et al., 2019). These were mapped using the ERIC online tool (*Strategy Design—The Consolidated Framework for Implementation Research* [available at https://cfirguide.org/choosing‐strategies/]) to identify strategies that may assist in enhancing the uptake of changing or selecting between PICC materials. Constructs whether identified as a barrier or enabler were inputted into the CFIR‐ERIC matching tool. As recommended by tool developers only level 1 strategy from the 73 possible ERIC strategies were reported (with all level 1 strategies ranking 50% or greater in strength of recommendation) (Powell et al., 2015).

**TABLE 2:** Matrix summary including related Consolidated Framework for Implementation Research (CFIR) domain, construct and statement theme findings.

CFIR domain	CFIR construct	Support statement subconstruct: Enabler (E) or barrier (B)	Exemplar quote (role, years of experience, transcript number)
Intervention characteristics	Intervention source (PICC material)	Limited perceptions of PICC material choices (B)	‘I've worked [with PICCs] … for 20 years … exclusively [traditional PICC], the only time we've really seen a variation of PICC is when there's a shortage of supply’. (Inserter, Purchaser, 15 years; 8) ‘traditionally they only put in one standard PICC here. It does come in a 4 fr and 5 fr. In terms of choice, … if their veins were a lot smaller or there was stenosis’. (Inserter, 5–10 years, 1) *[factors for selection of PICC material]* ‘patient history, medical background and any adverse events …. Especially if the study finds that the [material B] PICCs have a lower infection rate or less clotting with the [material A] … yeah, it would be useful to know the patient background before choosing a PICC to insert, as well as size [vessel size]’. (User, 11 years; 16) ‘Over that time yes, I have seen significant changes. The product is really different. The material the device is made of is really different to what it was 24 years ago. The way we use the PICC is different… the types of patients that have these devices inserted has also changes. I think probably we see more patients having PICCs inserter earlier in their medical journey … when I first starting nursing PICCs weren't that well utilized’. (User, 24 years, 14)
		Awareness of PICC material benefits (alternative PICC materials) (E/B)	‘A PICC that was more likely to not cause thrombosis would be beneficial. It would probably give us more confidence to put a bigger PICC in a smaller hole, … I can't see any reason why we wouldn't want to do that if we had a choice. Then, the [material B] is similar … it wouldn't affect us at all in our insertion, but—and we don't get to see the downstream effects of our insertions, but I would assume some of our PICCs get infected. Obviously having one that has an [material B] coating would have to be beneficial’. (Inserter, 11 years; 4) ‘With some of the devices, the actual gauge size is bigger which potentially could skew some data in that if … putting in a single lumen PICC. So we always go to a double… infrequently a triple. So I suppose if we know that one of those devices has a bigger gauge and it's not something that's [material A], then I suppose that in itself creates some issues because we could be getting a DVT simply because we have two lumens and they've got really arduous veins or poor vessel health’. (User, 15 years; 8) ‘If they've come in and they have … previous clots, if they've come in and they've had the port removed because it was infected … they've had a few days of antibiotics and then we put in a PICC temporarily … those [material B antimicrobial] PICCs to decrease the seeding of another infection’. (User, 11 years, 12)
		Vascular access device or service delivery rather than PICC material (B)	‘I think if [a dedicated/nurse‐led‐insertion] then nurses would feel empowered to opt for … an [material A, material B] PICC … if the outcomes are favourable … yes, it's $30 more if that's the cost difference, but the risk of this patient getting an infection is so significant, we're going to explore that. What's interesting is the patients that are high risk, they don't have—and because we probably haven't put [material B] in them, so thinking about like our home parental nutrition patients, we then add extra layers’. (User, 15 years; 8) ‘As in an infection risk? … I think it's time to get that infection under control in the body because some of the ones have turned into blood stream infections. A PICC is easier to take out [compared to a port a cath] … A PICC is a lot easier, less invasive’. (Inserter User, 6 years; 2)
	Evidence, strength, quality	Perceptions understanding of evidence (B)	‘consider alleged AMR [antimicrobial resistance] associated with the use of some coating/impregnation e.g. [material B] … we looked at the data and there wasn't overwhelming data to support it's use’. (Inserter, User, 11 years; 21) ‘In regard to things like thrombosis … if they have a higher incidence or a higher risk with thrombosis, that's going to really depend on what the outcome of the trial or current trials are. I know [material A] does make the assertion that they have anti‐thrombotic properties, but obviously that has to be evidence based, … currently with the current evidence the PICCs that we do choose is very dependent on what stock we have available’. (Purchaser, 11 years, 9) ‘Is it important that we change practice in line with any evidence which might … suggest that there may be choice in PICC selection, so if the evidence suggests there are options, [change] Yeah, we should change … keeping up with practice. Sometimes I feel like we're a little behind the ball with these kinds of things’. (Inserter, User, 6 years, 2).
	Relative advantage	Familiarity/availability priority over material advantages (B)	‘Once I have a look at the vessels and I've made my decision about certain PICC size, I'm not sure I've paid too much attention to the material. It's whatever's in stock … available then I just use that’. (Inserter, User, 11 years; 21) ‘In the past 10 years we've inserted [traditional] with a built‐in antiflow valve … So, we're quite familiar… we've got a team of probably eight to 10 inserters and we're all very happy with [standard care] … from an insertion point of view, but, we have not had any major problems with either of the [material A] or the [material B] PICC. From our perspective they're all okay—not that much difference. Some—there are some advantages with them and some disadvantages with them’. (Inserter, 11 years; 4)
		Material advantage for reduced complication and ‘kink‐memory’ (E/B)	‘I'm quite happy with the [material A]. It seems to do well. When they go really well, we've seen [patients] with a [material A] for pulmonary and hypertensive … for more than two, 3 years. So when we see the good ones that are really well looked after’. (Inserter, User, Purchaser, 11 years; 18) ‘based on a small pilot trial, the [material A] PICC … showed that the complications during dwell were reduced when that type of PICC was used. For some PICC choices we don't have that choice because that type of PICC isn't manufactured in [size or lumen number]’. (Inserter, 11 years; 22) ‘We insert [traditional] PICC and it's really easy to nurse… similar line to the [material A] PICC … because of the flexibility and the memory that it has. So if you do dress it [material A] wrong you can go back and fix the dressing and there's no issue with patency of the PICC. If you dress a [traditional] wrong it gains a kink memory and it becomes incredibly difficult to nurse … It doesn't matter how you dress it post that; it will always have that kink’. (Inserter, Purchaser 6–10 years; 3).

	Design quality and packaging	Product benefits related to brand familiarity rather than material or items in insertion kits (e.g. wire) (E, B)	‘currently our [traditional] PICCs that we've got are 50 cm long… good for 90% of patients, but, it's nice to have the slightly longer PICCs. Both the [material A] or the [material B] are 55 cm, or at least in part of the trial they've been 55. That's beneficial for taller patients. Sometimes we almost can't get as far around into the right side of the heart as we want, because we simply don't have the length of PICC. So, that's definitely beneficial for [material A] and [material B] PICCs’. (Inserter, 11 years, 4) ‘an example, the [material A] PICC that we were trialling was extremely sticky, and the kit was—[it] came with a lot of stuff that we couldn't use and didn't use’. (User, 7 years; 11) ‘so the [traditional], it has a muscle memory. So once it's got a kink in it, if that kink is there for a period of time, you can't unkink it’. (User, Purch 15 years; 8)
	Adaptability and trialability	Consistency of knowledge promotes consistency of education/practice (E/B)	‘The [alternative A] we'd actually been using intermittently … so I was familiar with it and … it's a little bit harder … slightly different … We always use the [traditional] that was already set up by predecessor who ran the department for 20 years … it was an easy to use PICC, reliable and we obviously moved away from the triple lumen because clot risk’. (Inserter, 11 years, 23) ‘So, we're quite familiar with the [traditional] product now, we've got a team of probably eight to 10 inserters and we're all very happy with the current [PICC] that we use, in—from an insertion point of view, but, we have not had any major problems with either of the [material A] or the [material B] PICC. From our perspective they're all okay – not that much difference’. (Inserter, 11 years, 4)
		Limited adaptability for intervention characteristics and brand loyal restricts selection (B)	‘[at the moment], with the world market… we are vey low on selection … I think industry is moving towards reducing their stock [with a variety of lumens] which is disappointing’. (Inserter, 11 years, 22) ‘For insertion, we use a [PICC guidance/positioning] technology [with PICC brand monitor] … so it's a special design… there aren't many PICCs out there that have that option’. (Inserter, 6 years, 2) ‘So knowing the ones that are currently available, I know that the [material B] ones, they can't be trimmed. So I guess trim‐ability would be another one [consideration for material selection]. The [traditional] ones you can trim, whereas the [material B] you can't’. (Inserter, 6 years, 2) ‘Yep. I think the trimming versus not trimming is—… with the [traditional and alternative B] PICCs that we use, their patented… soft end which [industry] alludes reduces the risk of vessel trauma because it's got that soft tapered tip … opposed to just a blunt end that does have the risk of causing some vessel trauma. So [traditional and alternative B manufacturer], would promote not cutting the tip because then you lose… technology. I don't really see any challenges in cutting versus not cutting outside of vessel trauma’. (User, Purchaser, 15 years, 8)
	Costs	Worthwhile if evidence for prevention (thrombosis, infection) (E)	‘… maybe that patient does need [material A] … if the outcomes are favourable. Or actually yes, it's $30 more if that's the cost difference, but the risk of this patient getting an infection is so significant, we're going to explore that. What's interesting is the patients that are high risk, they don't have—and because we probably haven't put [material B PICC] in them, but then we then add extra layers [dressings]’. (User, Purchaser 15 years; 8) ‘That could be as simple as these type of patients [ICU], unless they have a [material B] allergy, would be ideal. Like spend the money upfront. It might be a little bit more expensive, but they end up with infection, we pull the PICC and we put another PICC in them. So we're not really looking at vessel health and preservation from the get‐go’. (User, Purchaser 15 years; 8)
Cost restrictions or costs versus clinical effectiveness (E/B)		‘Unless of course it came down to maybe one—the other two PICCs were showing a huge failure rate, and one had maybe like a 5% failure rate and it was costing the hospital way too much. In that case, the health econ side of things, management would say, we're doing this from a costing point of view, it's costing the hospital too much to have these PICCs. So we need to have these certain PICCs’. (User, 7 years; 11) ‘For [patients] with a long‐term course of treatment, then they would have come back and get a second PICC, which obviously then just doubles the costs’. (User, 7 years; 11)
Outer setting	Patient needs and resources	Specific populations could benefit (E/B)	‘If a single ventricle [patient] gets clots in their upper venous circulation that precludes them from having their further cardiac surgical procedures, so it's a very big deal’. (Inserter, User, 11 years, 21) ‘antimicrobial's important, but I think less important, and thrombogenic is certainly incredibly important, particularly in our smaller patients’ … ‘Then it's something that's easy to use, that is comfortable and—you know, for the patient, so there's some patient satisfaction around it’. (Inserter, Purchaser, User, +11 years, 18) ‘if you had a patient that was more prone to infection, you might choose a antimicrobial PICC or if you had evidence of a patient being prothrombotic, like your cardiac or oncology patient population … we don't currently include patient's physiological factors into our decision making about PICC material’. (Inserter, Purchaser, +11 years, 22) ‘the person's thrombotic factors should probably be considered since we've been trying to reduce one of our major complications which is thrombosis’. (User, 11 years, 12)
		Individual patients and family unit may benefit (E/B)	‘Insertion is very small part of the PICC line for a patient’. (User, 7 years, 10) ‘the family and the patient want to be able to go home and complete their treatment at home, if and when possible and they don't want to have to come back to hospital because they have an infection or they have a swollen arm, or their catheter occludes. So, they want to know that they can go home and have uninterrupted care and they want to have a device that works while they are in hospital, that doesn't cause any pain or any additional treatment’. (Inserter, Purchaser, +11 years, 22)
		Patient journey less important than inserter experience (B)	‘For many, the operator preference is, you would say, is probably more important than the patient factors in their decision making’. (User, 7 years, 10)
Inner setting	Culture	Expert group to support decisions and practice needed (E)	‘What I am saying, is there's lots of technology in the vascular access world that doesn't translate to practice. I think we go with what we know works and perhaps that's not always the best option for the patient’. (User, Purchaser, 15 years, 8) ‘… I have done a lot of research … into nurse led service around ultrasound guided cannulation. Because … if we start there—if we can't have PICCs, maybe start with that for our … difficult Intravenous access patients. The literature indicates that a nurse led service is the way to go because—and our director here always advocates for this, as nurses are very good with process flow as a whole. Whereas our medical colleagues can sometimes go a little bit rogue. So I think when we look at a nurse led service and dedicated clinicians who are experts in vascular access, it makes sense. I think that would eliminate a lot of the challenges within—in the vascular access space for our patients and delay to treatment which is obviously an issue. Interestingly … we're actually seeing less PICCs inserted in this facility’. (User, Purchaser, 15 years, 8)
		Ideal does not occur in real‐world setting (B)	‘Yeah, just like … The [material A] PICC that we were trailing was extremely sticky, and the kits was (it came with a lot of stuff we couldn't use and didn't use) …. Whenever it came time to getting of the trial patients, when it was an [material A] PICC … for the inserters, always a groan or a grumble and not very happy about it …. It's always a bit more tricker to get in. Even with the patients that may have really great veins, maybe our [traditional] PICC would've just been a very easy straightforward PICC, sometimes it can just add … trickiness to the insertion’. (User, 7 years; 11)
	Leadership engagement	Management involved (E)	‘it should come from actual the department leadership, and then filtered down, as well as the radiographers, nurses and doctors’. (Inserter, User, 10 years, 1) ‘So with the [unit name] PICC inserting, not [medical imaging], it was the manager and we had I guess one person in the group of PICC inserters that had the leadership role/hat and they were the ones that really drove the change for when we started midlines … kind of like a—what do you call them—a champion, one champion in management’. (Inserter, Purchaser 6–10 years; 3)
		Clinicians inserting and using PICCs are engaged but have fixed ideas (E/B)	‘So it would be myself and I've got a small carde of hard core PICC inserters … and that's who it would be led by’. (Inserter User, 6 years; 2) [who should lead intervention change?] ‘I would be saying a lot of things but I think we would—rather than devices, our biggest issue is access at [hospital name], so we want more room time, we want patients to be able to get PICCs within 12 h of request, that would be our pie in the sky, it would be more about access rather than devices [material choice]. So, that would be really what our optimum service would be here in radiology, but we have a lot of barriers with staff, patient factors and room time would be our biggest issues’. (Inserter, 11 years, 9)
		Differing opinions‐specific inserters (E/B)	‘In the climate of our hospital, I think it would probably be one of the anaesthetists, with backing from our service, but one of the anaesthetists who is a lead for our service, who has a voice among their colleagues, the anaesthetic colleagues, and with backup from us’. (User, 24 years, 14)
	Implementation climate and relative priority	Change is hard and slow (B)	‘Education, especially at the bedside and ongoing is pivotal’. (Inserter, User, 6 years, 2) ‘I've seen that's happened a lot in this trial because we have such a huge rotation of junior doctors that get trained from the senior doctors, and get trained by the ones that may have a very strong opinion about certain products. … Nurses, especially nurses that are very comfortable in a position are always a bit hesitant towards change. But eventually after a year or 2 years once everyone's a little bit more comfortable with the product, generally that hesitation goes away and people just adapt’. (User, 7 years; 11)
		Key opinion leader ideas are more relevant than change (B)	‘discussion with the hospital executive team, in conjunction with interventional radiologists, because they have a lot of latitude in the products… and within their department with their experience for the products used. I guess it does defiantly come back to infection control, infection control has a lot of weight … as far as conducting literature review and using evidenced‐based practice to best guide the specific products, and what generally the wider healthcare community have found in their experience as ideal products to use, it's a very tricky thing to negotiate’. (Inserter/User, 7 years, 5)
		Intervention success enabled by support/education (E)	‘having a coordinated approach throughout the hospital for PICC selection, a tool which is common throughout the entire hospital or health service and even the state would bring consistency. That has to be evidence‐based, and I think that'll certainly help’. (In/Us, +11 years, 21)
	Networks and communications	Limited communication and networks for device placement/selection—material choice lower priority (B)	‘I think it comes down to funding in the end, I think. Because like I said, I'm aware of PICC lines just being done obviously after all the elective cases. Unfortunately, if [department] gets emergencies, it just pushes everyone else back. The patients and the team will just have to wait until a PICC line's put in. So, unless we get evidence to say how many people—I guess it's evidence based on if we get a PICC line in for a patient, we will actually help prevent bed blocking, so will it get earlier discharges? …, I guess maybe if we get funding and have an extra separate PICC line service maybe associated with [vascular surveillance department] … have a complete other service. I think that would really help change the [hospital name]’. (User, 6 years, 6)
		Networks valued when available (E)	‘it's got to start with [department] governance, because it's at the discretion of the [inserter discipline], and obviously infection control as well. Specifically, [Vascular Access Surveillance Team], play a huge role in the monitoring and surveillance of not only the PICC lines themselves, but also the documentation that goes along with them’. (In/Us, 7 years, 5)
Characteristics of individuals	Knowledge and beliefs about the intervention	Unique properties for specific PICC materials (B)	‘So we insert the [traditional] PICC and it's really easy to nurse. It's probably a similar line to the [material A] PICC that's easy to nurse as well because of the flexibility and the memory that it has. So if you do dress it wrong you can go back and fix the dressing and there's no issue with patency of the PICC. If you dress a [traditional] PICC wrong it gains a kink memory and it becomes incredibly difficult to nurse that along. So if someone's got large arms you've just dressed it wrong and it ends up in a crease and it kinks. That's kind of like you've killed that lumen if it's a dual lumen or you've made the single lumen quite difficult and sluggish to work and whatnot. It doesn't matter how you dress it post that; it will always have that kink’. (Inserter, Purchaser 6–10 years; 3) ‘A PICC that was more likely to not cause thrombosis would be beneficial. It would probably give us more confidence to put a bigger PICC in a smaller hole, … I can't see any reason why we wouldn't want to do that if we had a choice. Then, the [material B] idea is similar … it wouldn't affect us at all in our insertion, but—and we don't get to see the downstream effects of our insertions, but I would assume some of our PICCs get infected. Obviously having one that has an antimicrobial coating would have to be beneficial’. (Inserter, 11 years; 4) ‘With some of the devices, the actual gauge size is bigger which potentially could skew some data in that if … putting in a single lumen PICC. So we always go to a double… infrequently a triple. So I suppose if we know that one of those devices has a bigger gauge and it's not something that's [material A], then I suppose that in itself creates some issues because we could be getting a DVT simply because we have two lumens and they've got really arduous veins or poor vessel health’. (User, 15 years; 8)
		Waiting for evidence (E/B)	‘Yeah, it really depends on the result of the trial. So having been around doing this for a long time, and putting in central lines for a long time, if I have a bias towards a product—I do think that [material A] is a good product. I'm not a big fan of [material Bl] lines. I think the rise of anaphylaxis to antimicrobials and anaesthetic practice is on the increase. I think we use too much [antimicrobials]—with some good evidence for insertion and skin protection, but the rest of the evidence, I don't know if it's truly great’. (Inserter, purchaser, 11 years, 18) ‘Actually, I think because this trial has been going on for a couple of years. … I'm finding certain staff are leaning towards certain PICCs, and saying, they do actually—especially the [material A], in terms of insertion, it does have a little [curve] and that was a little bit of a challenge to begin with. But now that they've put in so many of them, they're finding, actually, this is a better PICC’. (Inserter, 5–10 years, 1)
		Familiarity priority over evidence (B)	‘… sometimes there's a certain brand that generally seems to work better, more often than not, and comes with‐ the wires exactly what we need, and if we troubleshoot, they're much easier to troubleshoot with …. It makes things defiantly much easier on the … when you're inserting because if there's problems with patient's vessels or anatomy or whatever, these easier‐to‐use PICCs makes things I guess easier for us to stick the PICCs in as opposed to a PICC that may tend to get sticky or get stuck, or comes with some wires that we can't use’. (Inserter, User, 7 years; 11)
	Self‐efficacy	What we practice is safe (E/B) and concerns are valuable evidence (E/B)	‘We have a fairly robust decision‐making process to determine the appropriate choice of PICC for the different indications’. (Inserter, 11 years, 22) ‘Yeah, I still find I'm a bit worried … It's still new, a new product as well, even we did a quite big number of studies. Like we haven't seen any reaction for the [material B] like severe reactions they talked about in the articles. It doesn't mean‐ what if it's happening in future practice’. (User, 7 years, 10) ‘We again, I guess from our point of view it comes down to you don't see these end results, so I don't see the patients that are‐having infections like a catheter‐associated blood stream infection so I'm not sure’. (User, 7 years; 11)
		Familiarity (E/B)	‘I think for the most part, and my knowledge as a PICC user, not an inserter, I think when PICCs are inserted by our team and those clinicians who are very familiar with PICC insertion, no, I think they do generally do a very good job of device selection. Sometimes, PICCs are inserted by those who are not as familiar, and maybe that's when we see those decisions that are maybe not as—the patient still receives a PICC. Maybe they got a double lumen PICC when maybe we should have looked at a single lumen only. But at the end of the day, that patient still got a PICC. They're still able to complete their treatment with that device, so it's not a complete loss, but maybe there could be some adjustments in those cases where the inserting clinician is not as familiar with our usual standard practices’. (User, 24 years, 14) ‘I guess you'd also look for—on the individual basis, you'd probably look for equipment you mostly [already knew] and are happy to use. Let's say if you've put in 6000 of one brand and there's a new brand that you haven't tried before and you haven't been trained on it, you would always use the equipment that you're more familiar with which you know you'll be—if you get any new equipment, you know there's a learning curve with them’. (Inserter, 11 years, 23)
	Individual stage of change	Rotation of staff or inexperience impacts on enthusiasm for innovation (E/B)	‘I've seen that's happened a lot in this trial because we have such a huge rotation of junior doctors that get trained from the senior doctors, and get trained by the ones that may have a very strong opinion about certain products …. Nurses, especially nurses that are very comfortable in a position are always a bit hesitant towards change. But eventually after a year or 2 years once everyone's a little bit more comfortable with the product, generally that hesitation goes away and people just adapt’. (User, 7 years; 11) ‘people who aren't necessary [vascular] access specialist, then there might be a learning curve to implement a new product’. (Inserter, Purchaser, +11 years, 22)
		Important and possible if aware of evidence or evidence clear (E)	‘Definitely. Why else would you do the research if you're not going to change your practice and we're a major tertiary hospital. I think it's the—if we had some good evidence and when we need to utilize it, then if we can improve care, why wouldn't we? Even if it is a cost thing, I think that the cost, say, of an infection, if that can be minimized, device replacement and vessel preservation is really the top of your—you know, because I've saved so much money not having to constantly replace devices due to failure and all of the palaver involved in that. I think the emotional stress of a child having to go into theatre is far outweighed by cost as well’. (User, 11 years, 12) ‘Currently, on our shelf we only have a four French and a five French [traditional]. So, we have two products, two PICCs, that's it. But there's no reason why we couldn't have three or four. We would—almost certainly—they wouldn't expire. We'd be—we do rotate through all those options pretty quickly, I would have thought…, why do we only have [traditional]? I'm sure it comes down to a cost or it's a hospital‐wide policy, I'm not sure, but I don't see any reason why we wouldn't be able to stock multiple vendors. I mean, we do It for other products’. (Inserter, 11 years; 4)
Implementation process	Opinion leaders	Related to familiarity bias or knowledge (B)	‘favouritism towards one, the [other] one will expire and again it's a cost benefit problem. I think we change from one and there needs to be a consensus from the majority within the department; if there's no benefit between the two, that we would have to pick one that would—everyone would be happy’. (Inserter, Purchaser, User, 11 years, 19) *[individuals for change, the roles]* ‘I think they're more like the doctors and the management, nursing management team. Yeah.… in the department. Nursing Unit Manager, that's what I think. They have positive belief of the product and stuff, they might put it forward. Yeah’. (User, 7 years, 10)
		Key stakeholders seem set on belief of product, being superior or inferior? (B)	‘Medical leadership taking up the baton and advocating for it and removing any product that wasn't evidence‐based’. (Inserter, Purchaser, +11 years, 22) ‘Nurses, especially nurses that are very comfortable in a position are always a bit hesitant towards change. But eventually after a year or 2 years … generally that hesitation goes away and people just adapt’. (User, 7 years; 11) ‘a coordinated approach helps in that if there is a lead for a particular hospital who can drive these across the various departments. One of the issues is that we all do our own thing in our own silos, and if they were to be a driver, a leader for this for a coordinated approach, I think that might help’. (Inserter, User + 11 years, 21)
	Executing and engaging	Education (E/B)	‘So I think it's about widespread education, both of like inserters, requesters, nursing staff looking after the PICC lines to make sure I guess that everyone understands those why they've been implemented, and how we can monitor and follow them up’. (User, 5 years, 20) ‘Okay, education. That would be a big thing. Also, no delays, like, let's say in another 3–4 years we're trying to implement this and the trial finished that long ago, no one will remember what it's all about. So, hopefully, the change will happen sooner. I think that will be a lot easier to implement. I just think education will be the number one’. (Inserter, 5–10 years, 1) ‘It will be everyone. As I said, the PICC team here are consistent of radiographers and nurses, so they'll both need that education. I wouldn't stress one over the other, I think they all [sort of] need that, yeah. In terms of doctors’. (Inserter, 5–10 years, 1) ‘Just a lot of education, a lot of positive—you might get people saying, this is crap, this is whatever. But saying, yeah, but look at the positives. Education on not just how to use the device but why it's better, why we've made the change’. (Inserter User, 6 years; 2)
	Reflecting and evaluating	Connected with trial outcomes (E/B)	‘I think just knowing what—I mean, we can probably look it up, but knowing what trials are happening in the area. Yeah, knowing that you're doing this [trial name] and … this is what you're looking at and then also closing that loop and finding out what the results were eventually once everything is published and analysed. Yeah, just knowing what is happening and what you are looking for and being a part of that instead of bringing in a change at the end’. (User, 14 years, 15) ‘[on reflection from the trial] Yeah, I think we would definitely need the education with different ones. If not, you're fumbling through it, you're just trying to pick it up as you go and we've got, you know‐….we'd need it to start that if the patient came from [insertion department], to have a little thing saying this is under trial, could you give feedback? If not, we're so quick on the ward and we're like, just do things because it's the way it's done. We're busy, we're an acute ward, we just keep going and we need to hand over from nurse to nurse so we need the education from the start’. (User, 18 years, 7)
		Ongoing feedback loop and education loop for innovation during trial and posttrial important (E/B)	‘There probably is. Probably a lack of … hmm … how to put it nicely? A lack of though around the actual device selection in most clinicians. The one big, clearly just because of the lack of education, I suppose. So there's a cohort that do selected devices, and you'd say were experts on central lines, or PICCs and then there's a—you know‐ 95% the other cohort that have an idea of how to put a PICC in, but perhaps not the device selection’. (User, 11 years, 12)

*Note*: PICC materials will be referred to as, traditional material, material A and material B.

Abbreviations: fr, french; PICC, peripherally inserted central catheter.

All five CFIR domains and constructs (e.g. intervention/individual characteristics, setting, implementation process) were considered. Outputs from the ERIC tool were then grouped into the nine clusters and were then aligned with practical real clinical examples, supported by methods from relevant literature to generate an enhancement plan/toolkit. Additionally, as ERIC is consensus‐driven tool, not all CFIR constructs have strong recommendations. Thus, the toolkit summary encompassed examples and practical suggestions drawn from literature, featuring five identified applied instances, while the remainder were derived from expert opinions.

### 3.8 Ethics

This study was conducted within the larger trial and approved by the health services and university's Human Research Ethics Committees (HREC/QCHQ/48682, GU 2019/094, UQ2019000446).

### 3.9 Reflexivity statement

The investigation team comprised of female researchers from nursing and physiotherapy disciplines with a range of educational qualifications from Graduate certificates to PhD, as well as researchers and clinicians who insert and manage PICC design selection. The team had a range of qualitative and research backgrounds, with senior and junior members, and individuals from both adult and paediatric healthcare backgrounds. None of the interviewers or participants had reported relationships with each other. The researchers acknowledge a potential bias of interest related to PICC material knowledge, but reported no authority or relationship with any participants, allowing for an open and honest discussion. We acknowledge that the investigation team likely represented early adopters and innovators (Berwick, 2003; Escobar‐Rodríguez & Romero‐Alonso, 2013) given the composition of clinician‐researchers, trialists and individuals; however, any bias was minimized by the use of CFIR to guide the interview schedule.

## 4 FINDINGS

### 4.1 Characteristics of participants

Out of the 50 participants contacted, 23 were interviewed. Seven participants primarily cared for children (<16 years of age), 15 primarily cared for adults and one participant had experience caring for both adults and paediatrics with PICCs. Two of the participants had five or fewer years of experience, seven had 10 or fewer years of experience, with the remaining half (*n* = 14) represented 11 or more years of experience (ranging from 11 to 30 years). Over half of the participants identified themselves as users (*n* = 12) and the remaining identified as inserters (*n* = 11). Of the 23 interviewees, nine participants reported an overlapping role, between purchaser and inserter or user and inserter, with no reports of purchaser alone. No repeat interviews were required and interview times ranged between 15 and 60 min.

### 4.2 Findings from corresponding CFIR domain

Participants answered questions about PICC insertion and material selection from all five major domains, with *intervention characteristics* and *inner setting* being the most populated (Table 2), and fewer responses in *outer setting* and *implementation processes*. Constructs were often discussed in an overlapping manner with a total of 16 constructs identified (additional excerpts are provided in Table 2). Findings are presented by domain [dominant construct(s) first] followed by less populated constructs; with overlapping constructs support statements and exemplar quotes aggregated. For the purposes of this evaluation, specific materials will be referred to as (1) traditional material, (2) material A and (3) material B.

#### 4.2.1 Intervention characteristics

The domain of *intervention characteristics* was heavily represented in the data generated. Key constructs in the domain included ‘intervention source’ with persistent referral to brand familiarity/loyalty and influence of tenders/purchasing contracts. There was also high‐level referencing to evidence‐based practice (‘evidence, strength and quality’) but detailed discussion about practice contraindicated this.

At the moment, because there's not good evidence for multiple types of PICCs, we don't have much of an opinion on material. Inserter, 11 years experience, interview no. 22

Other participants would refer to ‘evidence’ or ‘researcher’ but could not cite specifics of a study or guideline. Reference to an ‘evidence base’ was generic. Some participants also referred to prior research (e.g. previous trials, pilot trials) and their perceived advantages and disadvantages. The limited ability to site‐specific or contemporaneous evidence sources demonstrated tensions between evidence and real‐world practice.

Another construct which participants spoke to was *relative advantage* which overlapped with *design quality and packaging*. Some participants suggested perceived preference or advantage with different PICC materials; with preferences mostly related to availability or familiarity. Numerous participants discussed favouritism related to PICCs unrelated to material design (antimicrobial/hydrophobic properties) such as insertion kits rather than material, for example, insertion ease (stiffness of catheter or wire), sheath, PICC length, ability to trim, number of lumens, ability to secure/dress, kink memory:

We insert [traditional] PICC and it's really easy to nurse … similar line to the [material A] PICC, [but easier] … because of the flexibility and the memory that it has. So if you do dress [material A] wrong, you can go back and fix the dressing and there's no issue with patency of the PICC. If you dress a [traditional] wrong it gains a kink memory … if someone's got large arms, you've dressed it wrong and it ends up in a crease … kinks …. It doesn't matter how you dress it post that; it will always have that kink. Inserter, Purchaser; 6–10 years experience, interview no. 3

Intervention *trialability* was evident on a small scale, with many participants reporting having had experience with at least two of the intervention devices. The construct of *adaptability* was discussed in relation to clinician/culture/familiarity with device rather than active implementation into the environment.

I've worked [with traditional PICCs] … for 20 years … exclusively, the only … variation of PICC is when there's a shortage of supply. Inserter, Purchaser, >15 years experience; interview no. 8

Another sub‐theme evident was ‘brand familiarity’ such as in a universal PICC insertions system that made change difficult due to pre‐existing practices and loyalty (e.g. one industry system drives loyalty through unique system compatibility such as Apple). This was exemplified through historical precedent or consistent practice/product over decades.

We're not beholden to purchasing—we don't have purchasing agreements with [supplier] and because out volumes [of PICC use] are so high, that's why they give us stuff; but we pay full price for everything. I think we get a printer for free, but the printer … it is absolutely woeful. Inserter, Purchaser 6–10 years experience; interview no. 3

Otherwise, participants appeared to perceive little difference between the material in the intervention types, with few instances of describing the actual intervention as *complex*, implying that PICCs were easy to use. Due to the nature of the intervention of comparison [PICC/catheter material], discussion of *adaptability* was minimal and inferred when discussing ‘trim‐ability’ (cutting to reduce PICC length). Participants also reported that adaptation (trimming) was limited due to manufacturer guidelines, even when patient factors (for children and adults) necessitated ‘trim‐ability’. *Cost* also appeared to be a driving factor for decision‐makers who prioritized expenditure negating‐specific *patient needs*, for consideration of up‐front costs versus holistic valuation of devices and outcomes.

If there's a cost benefit for one or the other … I think it would be remiss for us to have both [more that one PICC] leads to … favouritism towards one or the [other]; one will expire and again it's a cost benefit problem. Inserter, Purchaser, User, 11 years experience; interview no. 19

I would—if they [decision makers] think this is a great idea or—the cost risk benefit is in the favour of the new device we would just move over [change practice]. Inserter, 10 years experience; interview no. 3

#### 4.2.2 Outer setting

The domain of *outer setting*, was discussed at a granular level with *patient needs and resources*. This specifically focused on the potential benefits and *patient needs* (antithrombogenic PICC for deep vein thrombosis or antimicrobial PICC for infection) when weighing up intervention versus potential complications.

[PICC] complications … lead to device replacement or lots of anti‐thrombolytic treatment … they're sold the PICC … ‘you don't need another needle again’ … and then saying ‘you're getting this needle [sub‐cut injections] because you've got the PICC in’, that can be quite confusing … for the [family] and the patients … I think it's stressful for the nursing staff … You know, inserting a PICC for their treatment and then we're stabbing them with a needle because they've got this PICC in. User, 11 years experience; interview no. 12

The discussion related to *outer setting* was dominated by device choice rather than design or material choice (aim of RCT). Many participants concentrated on the advantages and disadvantages of PICC devices compared to other vascular access devices (e.g. totally implanted devices) or optimal patient resources (refer to *inner setting, Section 4.2.3*). Additionally, participants described patients on the waiting lists, requiring multiple interim PIVCs instead of a timely PICC insertion, highlighting cost‐effectiveness, staffing limitations and suboptimal patient care.

Unfortunately, we don't have a separate PICC department … they filter out who's best in best serve [PICC insertion] … who is most appropriate depending on if there's a time gap in‐between cases … User, 11 years experience; interview no. 6

The absence of a dedicated PICC department/insertion model spoke to the *cosmopolitanism* construct, but comparison with other institutions was rarely explicit. The construct of *peer pressure* further demonstrated the desire for a nurse‐led or dedicated VA service for some. Additionally, the regular mentions of brand familiarity and loyalty (mentioned in *adaptability and trialability*) demonstrated *external policies and incentives*. One finding unrelated to pre‐defined constructs was the mention of challenges with device material complicated by local and global stock shortages, particularly worsened during the COVID‐19 and post‐pandemic periods.

I think currently with the current evidence, the PICCs that we do choose is very dependent on what stock we have available. Purchaser, 11 years experience; interview no. 9

#### 4.2.3 Inner setting

The domain of *inner setting* was discussed in the context of *structural characteristics* and *culture*. As discussed in CFIR (2009), there was a significant overlap of constructs. The organization of PICC insertion and care, whether as a service or through individual proceduralists (medical and/or nursing) shaped the perception of the intervention incorporation into individual practice This was especially clear when inserters were discipline‐specific (Interventional Radiologist vs. Nurse) but also related to purchasers (see *costs*). Service demand and device prioritisations (e.g. PICC service and PICC choice a default) had the largest impact on service and facility outcomes, rather than PICC material. More specifically, if a PICC insertion was not seen as part of the core business (e.g. insertion embedded within a larger department competing with other procedures) then patients waiting for PICC placement added burden on the system.

I think it's just people on the ward now being aware the delay in PICC lines … if we think about the patients in the end and … having a lot of patients needing PICCS and they obviously don't have the staffing or the time. User, 6 years experience; interview no. 6

The concept of PICC placement burden was contrasted with dialogue where PICC insertion/management was embedded and seen as the benchmark of quality vascular access care. The remaining constructs within the *inner setting* followed from *structural characteristics* and culture, rather than being independent. Statement(s) related to *leadership* (key influencers) were mostly related to service level PICC choices rather than patient level selection; depending on insertion service structure and some interdisciplinary preferences/differences. The construct of *implementation climate* was mentioned indirectly related to *priority for change* when discussing key stakeholders level of engagement and interest. *Tension for change* including the *priority for change* was related to perceived patient need/deficit for device rather than material selection. Lastly, *networks* and *communications* were valued; however, appeared to occur only at facility level or within specialized vascular access teams rather than hospital/service‐wide. There was no evidence of state‐wide, population level (adult or paediatric), speciality level (cancer care, etc.) or national practice consensus or networking.

#### 4.2.4 Characteristics of individual

Interviewed individual's reported *knowledge and beliefs* for *the intervention* (internally inconsistent) and *self‐efficacy* mostly overlapping with *familiarity*. Other participants alluded to preferences for PICC characteristics based on experience that informed their self‐beliefs. For example, a preference for a single‐lumen PICC, as it was as cheap as a double‐lumen PICC, or a double‐lumen PICC was inserted ‘just in‐case’ of clinical necessity. Contralaterally,

When PICCs are inserted [with] clinicians familiar with PICC insertion … they do generally do a very good job of device selection. Sometimes, PICCs are inserted by those [less] familiar, and maybe that's when we see those decisions that are maybe not as [ideal]—the patient still receives a PICC. Maybe they got a double lumen PICC when maybe we should have looked at a single lumen only. User, 24 years experience; interview no. 14

Then, is it also ease‐of‐use for it as well. So there is devices that I prefer to use over others, just for familiarity of using them commonly inside that. Having the ancillary equipment to make the insertion a bit easier … Insert, Purchaser, User, 11 years experience; interview no. 19

These sorts of preconceived beliefs have the risk of influence or bias when making PICC material selection. Other participants reported unique properties for specific PICC materials, such as kink‐memory.

We insert the [traditional] PICC and it's really easy to nurse. It's probably a similar line to the [material A] PICC that's easy to nurse as well because of the flexibility and the memory that it has. Inserter, Purchaser 6–10 years experience; interview no. 3

Overall, many individuals were sceptical about devices not standardly used with preconceived bias. Of note, some of these hesitations were related to broader PICC management and the health service rather than PICC materials. Participants reported superiority and safety *knowledge and beliefs* for bedside PICC insertion versus medical imaging which demonstrated a divergence of practice agility. This was underscored by further *knowledge/belief* statements that PICC insertion versus other vascular device, was not seen as an active choice, but rather a last resort. There were many statements about product performance and properties; some experience‐driven, and others urban myths. Interview participants mostly referred to *self‐efficacy* in relation to prior experience and training.

For the PICCs, we routinely put in the [traditional] ones all the time in medical imaging, so we absolutely are completely familiar with these and, yeah, we're keen on them. I didn't mind the one with the [material A] either. Inserter, 5 years experience; interview no. 17

Furthermore, despite participants representing diverse and complex experiences and expertise, trial results were dependent on *individual state of change* as many were sceptical of products until they reviewed trial results or specific excuses (e.g. product expiration) minimized willingness.

We probably then look at both devices in a trial phase … If there's a cost benefit for one or the other, that would need to then be factored in … [unlikely to keep both PICCs] ongoing, because that then leads to … favouritism towards one, the other will expire and it's a cost problem. Inserter, Purchaser, User, 11 years experience; interview no. 19

Few participants appeared to be *early adopters* or with limited knowledge of what was adopted elsewhere (Chopra et al., 2017); and most invested in preserving the ‘status quo’. Some participants were interested in seeing trial/emerging data, but ultimate considerations were related to cost and key decision‐makers choices (rather than PICC users or managers). Interestingly, participants unanimously described that large number of individuals, from differing levels of care, and PICC moments (insertion, management and surveillance) were required for change; but most importantly, repeated education was needed to initiate and support that change.

#### 4.2.5 Implementation process

Interview participants confidently reported that *key stakeholder* engagement (as reported in the *individuals‐involved* domain) played a vital role in the research and/or *implementation process*. Key stakeholders worked in a variety of disciplines (nursing and medical) and participant groups (inserter, manager and purchaser) across sites.

Many participants reported vascular access management services as the key group to leading policy and quality at the health service change; compared to end users (key/experienced inserters). Additional key stakeholders discussed included novice end users, key management *leaders*, patient populations (oncology and parents), industry, and interdisciplinary users; with importance placed on successful implementation requiring unit/service champions (individuals dedicated to supporting or driving change) (Damschroder et al., 2009).

The *executing* implementation had repeating elements of comprehensive approach/messaging; repeated, sustained, consistent information and education which embedded trial results penitent to PICC inserters and users. Interestingly, discussion around data (evidence) and *reflection/evaluation* of implementation was stated as useful but not important enough to make or initiate change; but requested to be provided in a timely manner. The larger issue of relative cost was often repeated by many (cost‐effectiveness) as a higher priority than evidence.

It would be good to have a central group … [vascular surveillance service] that they could give education … [and] a central point for escalation, education, for trials … so that you've got a point of contact for a person to say, we would like some education on this, or a new dressing, that we've got one person. User, 18 years experience; interview no. 7

This fed on from challenges for many in the current feedback loop (missing connections), despite the fact that all three hospitals had vascular access management and surveillance teams or committees. This demonstrated that despite the service, not all end users were aware of reports and internal structures for review.

### 4.3 Findings from CFIR–ERIC mapping

The 16 CFIR constructs were used for prioritization and mapping if they represented an enabler, barrier or both to PICC implementation and innovation. Most strategies were represented in the *implementation process* or *inner setting* and Domains (see Table 3). All five domains were represented, but the *characteristics of individuals* were only identified as a barrier, thus demonstrating no current enablers in that domain. A total of 15 strategies emerged, with the top four strategies identified were within *intervention characteristics* and *outer setting* comprising of promoting adaptability, accessing funding, obtaining consumer feedback and involving patients/consumers and family members. These 15 strategies were matched to six (Waltz et al., 2015) clusters to develop an enhancement plan, with three clusters not emerging (inferring no barriers) and were subsequently used to develop the implementation of the tool kit recommendations in Table 4. The tool kit summary was informed from the literature with five applied examples identified and the remaining developed by the expert opinion/authorship team. Tool kit items included established frameworks or methods such as the readiness assessment for change (e.g. organizational readiness to change assessment) (Helfrich et al., 2009) or Implementation Guide for Surveillance of CLABSI (2016) for auditing and feedback provision. Items of the toolkit without examples or existing methods, such as consumer engagement networks and local needs assessment were derived from expert opinion.

**TABLE 3:** Summary enablers/barriers for PICC material selection.

CFIR domain	CFIR construct barriers	CFIR construct enabler	CFIR construct barrier	Level 1 ERIC strategy^a^ (% of agreement for greater than 50% recommendation)
Intervention characteristics	Adaptability	✓	✓	Promote adaptability (73%)
	Cost		✓	Access new funding (72%)
Outer setting	Patient needs and resources	✓	✓	Obtain and use patients/consumers and family feedback (76%)
			✓	Involve patients/consumers and family members (71%)
			✓	Conduct local needs assessment (57%)
Inner setting	Implementation climate		✓	Assess for readiness and identify barriers and facilitators (52%)
	Culture	✓	✓	Identify and prepare champions (52%)
	Networks and Communications	✓	✓	Promote network weaving (57%)
				Organize clinician implementation meetings (52%)
Characteristics of individuals	Knowledge and beliefs about the intervention		✓	Conduct educational meeting (56%)
Implementation process	Opinion leaders	✓	✓	Identify and prepare champions (64%)
	Key stakeholders		✓	Identify and prepare champions (63%)
	Reflecting and evaluating		✓	Develop and implement tools for quality monitoring (60%)
	Opinion leaders	✓	✓	Inform local opinion leaders (57%)
	Reflecting and evaluating		✓	Audit and provide feedback (56%)

Abbreviations: CFIR, Consolidated Framework for Implementation Research; PICC, peripherally inserted central catheter.

^a^ Expert Recommendations for Implementation Change (ERIC) strategies may enhance the uptake of changing or selecting between PICC materials.

**TABLE 4:** Implementation enhancement plan/toolkit (ERIC mapping) enablers and barriers.

ERIC cluster (Waltz et al., 2015)	ERIC strategy (Powell et al., 2015)	Definition (Powell et al., 2015)	Examples (applied suggestions)
Use evaluative and iterative strategies	Assess for readiness and identify barriers and facilitators	‘Assess various aspects of an organization to determine its degree of readiness to implement, barriers that may impede implementation and strengths that can be used in the implementation effort’	Survey leaders and/or clinical end users with specific tools for readiness assessment for change [e.g. organizational readiness to change assessment (ORCA) (Delaforce et al., 2023) or Dimensions of Organizational Readiness‐Revised (DOOR‐R)]
	Audit and provide feedback	‘Collect and summarize clinical performance data over a specified time period and give to clinicians and administrator to monitor, evaluate and modify provider behaviour’	Use local surveillance teams and incidence systems and with surveillance frameworks PICCs [e.g. Implementation Guide for Surveillance of Central Line Associated Blood Stream Infection (CLABSI) (2016)]. Surveillance Initiative Publications | Australian Commission on Safety and Quality in Health Care
	Develop and implement tools for quality monitoring	‘Develop, test and introduce into quality‐monitoring system the right input0 the appropriate language, protocols, algorithms, standards and measures (of process, patient/consumer outcomes, and implementation outcomes) that are often specific to the innovation being implemented’	Codesign with facility clinicians and moderators gaps in service provision tools (protocols, meetings, etc.), quick reference education guides for alternative PICC materials/design choice [e.g. The Michigan Appropriateness Guide for Intravenous Catheters (MAGIC) (Ullman et al., 2020) or The Michigan appropriateness guide for intravenous catheters in paediatrics: miniMAGIC] (Ullman et al., 2019)
	Obtain and use patients/consumers and family feedback	‘Develop strategies to increase patient/consumer and family feedback on the implementation effort’	Survey patient experience, PROM/PREM with specific focuses, advertise/identify consumer representative(s) with lived PICC experience or collaborate with consumer organizations for high end users requiring PICC treatment provisions (e.g. Parenteral Nutrition Down Under (pndu.org) or Cystic Fibrosis Australia—Embracing Tomorrow)
	Conduct local needs assessment	‘Collate and analyse data related to need of intervention’	Review PICC insertion model and surrounding supportive services
Adapt and tailor to context	Promote adaptability	‘Identify the ways a clinical innovation can be tailored to meet local needs and clarify which elements of the innovation must be maintained to preserve fidelity’	End user and industry co‐design for specific PICC innovation (e.g. insertion kits) If considering innovation related to eHealth or documentation, perform a clinical workflow analysis to support the implementation of eHealth interventions including identifying discrete workflow components, workflow assessment, triangulation and stakeholder proposal of intervention implementation (Escobar‐Rodríguez & Romero‐Alonso, 2013)
Utilize financial strategies	Access new funding	‘Access new or existing money to facilitate the implementation’	Scope populations that use PICCs the most and work with charitable organizations to improve care delivery outcomes for those groups who need PICCs the most
Develop stakeholder relationships	Identify and prepare champions	‘Identify and prepare individuals who dedicate themselves to supporting, marketing and driving through an implementation, overcoming indifference or resistance that the intervention may provoke in an organization’	Identify staff members in all levels of health care with specific focus on departments that work with PICCs (insertion, purchasing and management)
	Inform local opinion leaders	‘Inform providers identified by colleagues as opinion leaders or “educationally influential” about the clinical innovation in the hopes that they will influence colleagues to adopt it’	Meet with healthcare executives, inserters/insertion teams with evidence for PICC material to promote accurate education
	Promote network weaving	‘Identify and build on existing high‐quality working relationships and networks within and outside the organization, organizational units, teams, etc. to promote information sharing, collaborative problem‐solving and shared vison/goal related to implementing the innovation’	Interest groups (e.g. State‐wide or national) and relevant professions societies (Association for Vascular Access‐Association for Vascular Access [avainfo.org]), National Infusion and Vascular Access Society (NIVAS | NIVAS), Association for Radiologic and Imaging Nursing (arinursing.org), Australian Society of Anaesthetists or Australian College of Critical Care Nursing ACCCN—Australian College of Critical Care Nurses
	Organize clinician implementation meetings	‘Develop and support teams of clinicians who are implementing the innovation and give them protected time to reflect on the implementation effort, share lessons learned and support one another's learning’	Form facilitation groups using published frameworks or examples (Berwick, 2003; Waltz et al., 2015)
Train and educate stakeholders	Conduct educational meetings	‘Hold meetings targeted towards different stakeholder groups (e.g. providers, administrators, other organizational stakeholders and community, patient/consumer and family stakeholders) to teach them about clinical innovation’	Hold a facility symposium open to the public with panel of experts, researchers, key clinicians and consumers to discuss improving PICC care (imbed PICC material topics)
Engage consumers	Involve patients/consumers and family members	‘Engage or include patients/consumers and families in the implementation effort’	Develop or identify patient information about the PICCs, ‘ask your health care provider more about this option’ sort of promotion

Abbreviations: ERIC, Expert Recommendations for Implementation Change; PICC, peripherally inserted central catheter.

## 5 DISCUSSION

Innovation in PICC materials and design is likely to be iterative, as technology, research and industry converge to build and apply new technologies. Although clinical trials are preferred, innovations are frequently assessed through laboratory studies, followed by post‐market surveillance to ensure safety (Ullman et al., 2019). However, this study has demonstrated that the clinicians, health services and key proceduralists (experts) have specific contexts that promote or prevent the implementation of alternative products into practice.

One dominant theme in implementation contexts revolved around evidence‐based practice, often presented as a broad concept. However, tensions arose between applying *evidence strength* and real‐world practice. While clinical trials, like the one underpinning this study, represent a stride towards implementation, additional evaluative and iterative strategies (e.g. quality monitoring and audit/feedback) are essential for progress. This is similar to previous studies supporting implementation of medication safety through utilization of audit/feedback (Davies et al., 2019) and quality monitoring for pressure injury prevention (Tayyib & Coyer, 2017). Another strong theme was the high level of brand familiarity and loyalty, which were tied to purchasing contracts, design quality, familiarity and packaging. Overcoming brand familiarity may require working with industry to consider redesign or adaptable options to meet clinician/consumer needs. Furthermore, the ERIC‐based implementation strategies recommended focus on the promotion of adaptability, *patient needs* and the integration of key stakeholders. These strategies are not unique to vascular access practice needs, and a combination of changes that incorporated *patient needs* and staff agility have been useful for intensive care redesigns (Patterson et al., 2019), and communication/handover frameworks (Mackie et al., 2021).

In this study, it was evident that the choice of PICC material played a secondary role within the broader context of PICC insertion decisions and vascular access device selection. Any focus towards PICC material appeared to be confined to historical preferences, self‐efficacy or the experience of individualized or specialized inserters. Higher priority items discussed by participants for PICC material selection were lumen numbers, catheter–to‐vein ratio, insertion process similarities and other elements (trim‐ability, kinking, insertion wire design and insertion kits). There was less focus on patient‐focused care delivery, with little mention of consumers if not prompted, matching with the need to strengthen *outer setting* strategies as identified in ERIC (Waltz et al., 2015). A limited number of participants appeared to have comprehensive *knowledge* about the entire life cycle of a PICC and its surveillance outcomes within their healthcare facility. For most, their familiarity was confined to the specific moment of care delivery (inserters: insertion, users: failure and patient impact, purchasers: cost and sustainability of product). Those individuals aware of potential benefits or consideration of the patient, were most often advanced practice inserters or senior members who were part of surveillance teams. This underscores the importance of alienating PICC material choices with broader healthcare delivery decisions until there is robust evidence integrated into policy. This is compounded by a delay of research translation to practice as seen in the continued use of heparinised saline for central devices despite low‐quality evidence (Lopez‐Briz et al., 2022). Therefore, the local context for implementation, PICC insertion and monitoring models is vital when considering PICC practice change and translation of trial results.

Clinicians isolated awareness of PICC moments, highlights the need to support networking (*inner setting*) and *implementation process*; also identified in the ERIC mapping. If purchasing a PICC is not a priority of health service delivery or is not aligned with of core objectives, the choice of PICC material becomes a secondary concern, potentially leading to *patient needs* or individualized healthcare taking a back seat. falling behind (Chopra et al., 2015; Schults et al., 2022; Ullman et al., 2020). This could be associated with a lack of organizational or regional (service or state‐based) opportunity to benchmark complication or failure rates to consider the need for innovation. Many inserter participants commented they did not know the exact failure rate, causes and contributing factors. Further, participants highlighted that there was a lack of feedback from past monitoring and surveillance in relation to prior PICC failure and innovation.

Feedback and review might be possible; however, the entrenched beliefs or self‐assurance of individuals regarding familiar PICC materials during insertion could serve as the largest barrier to future change. Interestingly, this study did not identify strong ties with external policy as a theme which may be unique to this study setting. This is in contrast to the dominant North American focus on CLABSI prevention and formalized structures for complications of vascular access (both thrombosis and CLABSI) (Milella et al., 2021). Therefore, as Damschroder et al. (2009) propose regarding Rogers' Diffusion of Innovations (innovators and early adopters vs. laggards) (Damschroder et al., 2009), decisions about PICC materials and related innovations need interdisciplinary consultation or collective assessment to address varying levels of confidence in the insertion process.

### 5.1 Implications and lessons from ERIC CFIR

The ERIC–CFIR mapping demonstrated that while barriers existed, strategies to overcome implementation challenges were reliant on local audit data and key stakeholders' readiness for change. Despite an innovative material PICC appearing straightforward practice change, due to its fixed nature throughout treatment compared to the insertion procedure, this study demonstrates more contextual challenges (see examples in Table 4). Fostering support within the *inner setting*, before, during and after the change, would be crucial for ensuring successful implementation. For example, engaging local key stakeholders in the development of quality monitoring tools and implementing continuous audits and feedback on PICC performance. Moreover, despite not identifying changes in infrastructure and interactive support, it was evident that funding sources were important when attempting to start and sustain changes to practice. Lastly, as most healthcare facilities do not have implementation experts as part of core clinician staff, this study also generated recommendations and a refined toolkit of implementation strategies, to optimize end of study translation of findings into policy and practice for use at local and wider facility locations post‐RCT result dissemination.

### 5.2 Strengths and limitations of the work

This work has limitations. In particular, it is based on a single country setting and inclusive of clinicians who were already influenced by the trial(s) in their service. It is possible that this participation influenced their opinions of alternative products. Additionally, all health services involved are Australian and publicly funded, so financial motivators in alternative funding systems (e.g. private) are likely to be significantly different.

Equally, this project has considerable strengths. It is a robust qualitative exploration, using high‐quality methods, that pragmatically focuses on the development of a product‐agnostic toolkit for implementation. The CFIR and ERIC strategies could easily be applied to many different products, as technology continues to evolve. Additionally, we included a variety of stakeholder opinions from across traditional disciplines from paediatric and adult settings, rather than focusing on the disciplines that have control over device selection and material in current healthcare settings.

### 5.3 Recommendations for further research

Since many of the alternative and traditional PICCs evaluated in the parent trial are utilized across health service(s), they could represent real, rather than perceived, barriers and facilitators. Exploring these aspects prospectively is crucial. Moreover, while patients and families were not interviewed in this study, their inclusion in future research could offer valuable insights into the impact of materials throughout the lifespan of both patients and devices. This would shed light on how materials affect patients and devices, benefiting industry and clinicians alike.

### 5.4 Implications for policy and practice

Understanding the intricate dynamics surrounding the choice of PICC materials within healthcare settings unveils pivotal implications for both policy and practice. The observed reliance on familiar products, influenced by historical preferences and individual beliefs, underscores the need for policy frameworks that promote the integration of evidence‐based decision‐making. Policy measures should support interdisciplinary consultations, acknowledging the varying levels of confidence among practitioners regarding new material adoption. Moreover, healthcare facilities should recognize the significance of funding sources for implementation (alternative PICC materials) and the need for financial support aligned with practice transformations. Incorporating patient and family perspectives within policy considerations, unexplored in this study, would provide a comprehensive understanding of material impacts across the patient and device lifespan, enlightening industry and clinicians alike. This holistic approach could shape policies conducive to fostering acceptance of innovative materials, ultimately enhancing patient care and outcomes within healthcare practices (Wang et al., 2019).

## 6 CONCLUSION

Clinical trial evidence is important, but in this area of healthcare decision‐making and practice, new evidence does not ensure practice change. It is vital that local contexts are considered, especially resourcing. Our study demonstrated the implementation contexts in the Australian health environment and has provided a practical toolkit of strategies that can be readily employed when implementing alternative PICC materials and designs. These must include evaluative strategies (e.g. audit/feedback from consumers and clinicians, local needs assessment) adaptation (e.g. tailoring insertion kits to meet local needs) and the development of key stakeholder relationships (e.g. local and network *opinion leaders*). PICC material and design will continue to innovate through the convergence of technology, research and industry often through health services‐focused research. Having a comprehensive understanding of clinicians and proceduralist‐specific contexts that dictate the adoption of evolving products and practice, thus underpinning the steps needed to bridge the evidence‐practice gap.

## AUTHOR CONTRIBUTIONS

The authors cited have made substantial contributions to the design (DA, RMW, VG, NM, TMK, AD, CM, AU and SK), acquisition of data (DA, RMW, VG and CM), interpretation of the data (DA, RMW, VG, NM, TMK, AD, CM, AU and SK), drafted the manuscript (DA, RMW, NM, AD, AU and SK) and revision for critical content the manuscript (DA, RMW, VG, NM, TMK, AD, CM, AU and SK).

## FUNDING INFORMATION

The authors received no funding for this study, however, this study was nested within a Grant from the National Health and Medical Research Council (APP1157178).

## CONFLICT OF INTEREST STATEMENT

DA reports speaker fees provided to her employer University of Queensland for NAVI technologies and 3M, unrelated to this project. RMW received research grant funding for this project and another investigator‐initiated research grant provided to Griffith University by vascular access product manufacturer Becton Dickinson and Company. NM reports Griffith University and the University of Queensland have accepted on her behalf investigator‐initiated grants from 3M, Eloquest Healthcare and Cardinal Health, unrelated to this study. TMK reports Griffith University and the University of Queensland have accepted on her behalf investigator‐initiated grants from 3M, BD, Smiths Medical and consultancy from Medical Specialties Australia unrelated to this study. AU reports investigator‐initiated research grants paid to her employer from BD and 3M, unrelated to this study. SK reports monies received by her employer QUT from BD Medical and ITL Biomedical for educational consultancies unrelated to this study. AD, VG and CM declare no conflicts of interest.

## PEER REVIEW

The peer review history for this article is available at https://www.webofscience.com/api/gateway/wos/peer‐review/10.1111/jan.16342.

## Supporting information


Table S1.

Table S2.

## Data Availability

Data available upon request.
