# Supplementary material for: Implementation contexts and strategies for alternative peripherally inserted central catheter material and design selection: A qualitative exploration using CFIR/ERIC approach
Source: J Adv Nurs. 2024 Jul 24;81(11):7067–86. doi: 10.1111/jan.16342 (PMC12535357; doi:10.1111/jan.16342)
Supplement: Supplementary file 1 — Table S1. [file JAN-81-7067-s002.docx]

Supplementary Table 1:Vascualr Access/Device Terms

| **Abbreviation or term** | **Definition** |
| --- | --- |
| **Antibiotic or antimicrobial agents** | Agent for decontamination or prevention of infection e.g. chlorhexidine |
| **CVR** | A ratio measurement of vein size compared to catheter (PICC diameter), known as catheter to vein ratio |
| **Champions** | Individuals dedicated to supporting or driving change(9) |
| **French** | Unit of measure or size of a catheter (abbreviated as fr) |
| **Hydrophobic** | Water repelling |
| **Implanted vascular access devices** | Long term central access devices placed entirely under the skin and are accessed through external needles (no exposed catheter, e.g. port a cath). |
| **Infection** | Within the context of a blood stream infection related/unrelated to PICC or other invasive device in the body |
| **Medical imaging/radiology services** | A department where radiology field practices, with the use of medical imaging technologies such as fluoroscopy, CT scan, etc, with PICC lines are often inserted as part of the department services |
| **PICC** | A vascular access device where the insertion site is peripheral but the tip passed through to the large veins near the heart, ideally tip located at , known as Peripherally inserted central catheter |
| **PICC department/service, VA service** | Speciality service for the insertion and/or maintenance/surveillance of PICC devices in the hospital or hospital region. May also be called vascular access service |
| **PICC inserter** | A clinician who is trained or recently placed PICC lines |
| **PICC manager or user** | A clinician who accesses or maintains PICC use including but not limited to maintenance near insertion to removal, dressing and needless connector changes, infusion of fluids/medications |
| **PICC purchaser or decision maker** | A clinician involved in the purchasing or selecting of possible PICC products used in a department; from moment of connection with industry, re-ordering of product or financial delegation to bring product into clinical area |
| **Thrombosis** | A blood clot inside a blood vessel |
| **Traditional PICC** | Polyurethane PICCs with no alternative material |
| **Trim (trim-ability)** | Cutting end of PICC to reduce length, capacity to trim PICC |
| **Vascular access device** | Sometimes called venous access devices, a small tube inserted for the provision of therapy (medications, fluids) for medical needs. They can be inserted peripherally or centrally |
