# Supplementary material for: Implementation contexts and strategies for alternative peripherally inserted central catheter material and design selection: A qualitative exploration using CFIR/ERIC approach
Source: J Adv Nurs. 2024 Jul 24;81(11):7067–86. doi: 10.1111/jan.16342 (PMC12535357; doi:10.1111/jan.16342)
Supplement: Supplementary file 2 — Table S2. [file JAN-81-7067-s001.docx]

***Supplementary Table 2: NAME***

| **Domain [question number]** | **Key Points and exemplar quotes** |
| --- | --- |
| **Inserter general [1]** |  |
| **Inserter evidence strength, quality and advantages [2]** |  |
| *Inserter evidence strength[2]* |  |
| *Inserter evidence quality [2]* |  |
| *Inserter evidence advantages [2]* |  |
| **Inserter patient needs and resources [3]** |  |
| **Inserter tension for change, priority for change, and leadership engagement [4]** |  |
| Inserter tension for change, [4] |  |
| Inserter priority for change [4] |  |
| Inserter leadership engagement [4] |  |
| **Inserter knowledge of benefits about intervention [5]** |  |
| **Inserter planning and opinion leaders [6]** |  |
| *Inserter planning [6]* |  |
| *Inserter opinion leaders [6]* |  |
| **Inserter other observations** |  |
| **User general [1]** |  |
| **User evidence strength, quality and advantages [2]** |  |
| *User evidence strength [2]* |  |
| *User evidence quality [2]* |  |
| *User evidence [2]* |  |
| **User patient needs and resources [3]** |  |
| **User tension for change, priority for change, and leadership engagement [4]** |  |
| *User tension for change [4]* |  |
| *User priority for change[4]* |  |
| *User leadership engagement [4]* |  |
| **User knowledge of benefits about intervention [5]** |  |
| **User planning and opinion leaders [6]** |  |
| *User planning [6]* |  |
| *User opinion leaders [6]* |  |
| **User other observations** |  |
| **Purchaser general [1]** |  |
| **Purchaser evidence strength, quality and advantages [2]** |  |
| *Purchaser evidence strength [2]* |  |
| *Purchaser evidence quality [2]* |  |
| *Purchaser evidence advantages [2]* |  |
| **Purchaser patient needs and resources [3]** |  |
| **Purchaser tension for change, priority for change, and leadership engagement [4]** |  |
| *Purchaser tension for change [4]* |  |
| *Purchaser priority for change [4]* |  |
| *Purchaser leadership engagement [4]* |  |
| **Purchaser knowledge of benefits about intervention [5]** |  |
| **Purchaser planning and opinion leaders [6]** |  |
| *Purchaser planning [6]* |  |
| *Purchaser opinion leaders [6]* |  |
| **Purchaser other observations** |  |
